# Diptoindonesin G promotes ERK-mediated nuclear translocation of p-STAT1 (Ser727) and cell differentiation in AML cells

**DOI:** 10.1038/cddis.2017.159

**Published:** 2017-05-04

**Authors:** Jian Gao, Minmin Fan, Gang Xiang, Jujuan Wang, Xiong Zhang, Wenjie Guo, Xuefeng Wu, Yang Sun, Yanhong Gu, Huiming Ge, Renxiang Tan, Hongxia Qiu, Yan Shen, Qiang Xu

**Affiliations:** 1 State Key Laboratory of Pharmaceutical Biotechnology, School of Life Sciences, Nanjing University, Nanjing 210093, China; 2 Department of Hematology, The First Affiliated Hospital of Nanjing Medical University, Nanjing 210029, China; 3 Department of Clinical Oncology, The First Affiliated Hospital of Nanjing Medical University, Nanjing 210029, China; 4 Collaborative Innovation Center of Chemistry for Life Sciences, Nanjing University, Nanjing 210093, China.

## Abstract

Exploration of a new differentiation therapy that extends the range of differentiation for treating acute myeloid leukemia (AML) is attractive to researchers and clinicians. Here we report that diptoindonesin G (Dip G), a natural resveratrol aneuploid, exerts antiproliferative activity by inducing G2/M phase arrest and cell differentiation in AML cell lines and primary AML cells. Gene-profiling experiments showed that treating human leukemia HL-60 cells with Dip G was associated with a remarkable upregulation of STAT1 target gene expression, including IFIT3 and CXCL10. Mechanistically, Dip G activated ERK, which caused phosphorylation of STAT1 at Ser727 and selectively enhanced the interaction of p-STAT1 (Ser727) and p-ERK, further promoting their nuclear translocation. The nuclear translocation of p-STAT1 and p-ERK enhanced the transactivation of STAT1-targeted genes in AML cells. Furthermore, *in vivo* treatment of HL-60 xenografts demonstrated that Dip G significantly inhibited tumor growth and reduced tumor weight by inducing cell differentiation. Taken together, these results shed light on an essential role for ERK-mediated nuclear translocation of p-STAT1 (Ser727) and its full transcriptional activity in Dip G-induced differentiation of AML cells. Furthermore, these results demonstrate that Dip G could be used as a differentiation-inducing agent for AML therapy, particularly for non-acute promyelocytic leukemia therapy.

Acute myeloid leukemia (AML) is a clonal hematological malignant disease of developing myeloid cells that is characterized by uncontrolled proliferation and a block in normal hematopoietic cell differentiation.^1^ To date, standard therapies used to treat AML have been cytotoxic agents that target rapidly proliferating cells. This therapeutic approach has limited efficacy and significant toxicity.^2^ The success of all-*trans* retinoic acid (ATRA) in the treatment of acute promyelocytic leukemia (APL), a distinct subtype of AML, has opened new perspectives for differentiation therapy.^3, 4^ However, ATRA-mediated differentiation therapy is not available for the other types of AML.^5, 6^ Therefore, novel and less toxic therapeutic agents that are capable of overcoming differentiation arrest are urgently needed for AML therapy.

Naturally occurring small molecules are an important source of drug leads. Diptoindonesin G (Dip G), a resveratrol (Rev) aneuploid, can be either naturally isolated from the stem bark of tropical plants such as *Hopea chinensis* or totally synthesized.^7, 8, 9^ Our previous study demonstrated that Dip G possesses immunosuppressive activities against activated T cells.^9^ A recent study showed that Dip G acts as a selective estrogen receptor modulator for the treatment of human breast cancer.^10^ Although Rev and its analogs can inhibit cell growth and induce apoptosis and differentiation in human leukemia cell lines,^11, 12, 13, 14^ the antileukemic properties of Dip G are still undefined.

The activation of signal transducer and activator of transcription 1 (STAT1) has a vital role in the terminal differentiation of immature leukemia cells. STAT1 activation was first identified in ATRA-induced myeloid differentiation and confirmed in various drug-induced leukemia cell differentiation.^15, 16, 17, 18, 19^ STAT1 activity is regulated by phosphorylation on tyrosine 701 by the Jak family members, important for its dimerization, translocation to the nucleus and binding to DNA.^20^ Phosphorylation of STAT1 at a second site (serine 727) in the transcription activation domain is regulated by the MAPK signaling cascade, including MEK, ERK, p38 and JNK, and is required for full transcriptional activity of STAT1.^21, 22^ Phosphorylated STAT1 migrates from the cytoplasm to the nucleus and transactivates its target genes, such as IFIT3 and CXCL10, to induce cell differentiation.^23, 24^ STAT1 silencing or phosphorylation-deficient STAT1 has been reported to inhibit the induction of AML differentiation.^17, 25, 26^

In this study, we revealed that Dip G could induce differentiation in AML cells. Unlike ATRA-induced classical differentiation, which increases STAT1 expression and its phosphorylation at both Tyr701 and Ser727, Dip G selectively drives the nuclear translocation of p-STAT1 (Ser727) and subsequently facilitates the transcription of differentiation-related genes. These findings shed light on the mode of action of a novel differentiation-inducing agent and provide a therapeutic candidate for the treatment of AML.

## Results

### Dip G inhibits AML cell proliferation

**Figure 1:**
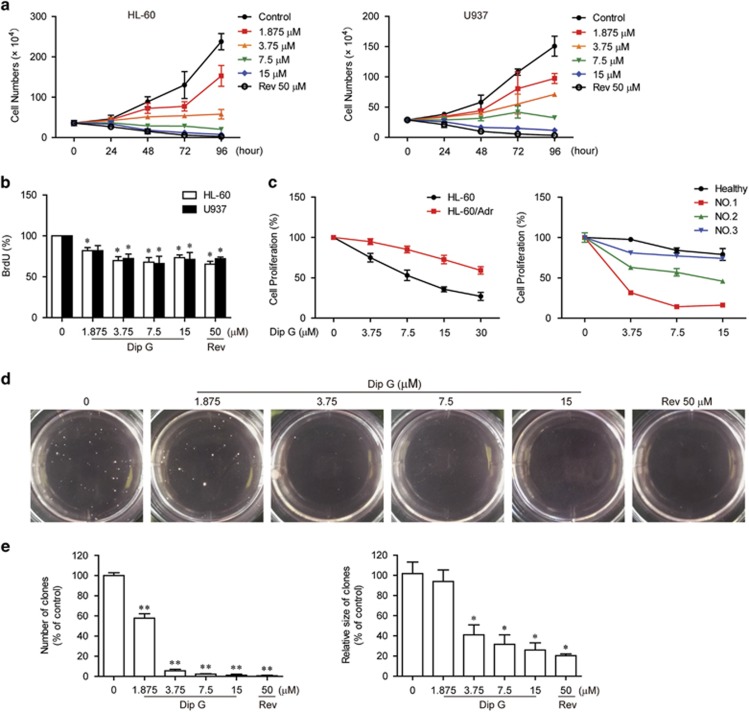
Antiproliferative effects of Dip G on AML cells. (**a**) HL-60 and U937 cells were treated for 96 h in the presence of the indicated concentrations of Dip G or Rev (50 *μ*M), and the number of cells were counted using a Trypan Blue exclusion assay. (**b**) Effects of Dip G on cell proliferation after 24 h treatment using BrdU incorporation assay. (**c**) HL-60, HL-60/Adr or primary AML cells were treated with various concentrations of Dip G for 72 h. The rate of cell proliferation was measured using a MTT assay. (**d**) HL-60 cells were pretreated with Dip G or Rev for 24 h. Then 5000 viable cells were cloned in soft agar and cultured for another 20 days. The six-well plates were photographed using a digital camera. The colonies >50 *μ*m in diameter were counted under an inverted microscope at × 40 magnification. (**e**) Statistical analysis showing the percentage of colonies (upper panel) and size (lower panel) relative to the control cells (control cells=100%). Data are shown as the mean±S.E.M. of three independent experiments. *P<0.05, **P<0.01 versus the control group without any treatment

Both HL-60 and U937 cells were exposed to Dip G and examined using the Trypan Blue dye exclusion method. Compared with the untreated controls, 1.875 to 15 *μ*M of Dip G greatly reduced viable cell numbers (Figure 1a and Supplementary Figure S1a). The reduction with 15 *μ*M Dip G was comparable with 50 *μ*M Rev. To determine whether the decrease of the viable cell density was due to the antiproliferative effect of Dip G, bromodeoxyuridine (BrdU) assay was performed to valuate cell proliferation. As shown in Figure 1b, the cell proliferation was significantly inhibited after 24 h of Dip G treatment. In addition, Dip G inhibited cell proliferation in a dose-dependent manner in the HL-60-resistant cell line HL-60/Adr and some primary leukemic cells from AML patients (Figure 1c). The antiproliferative activity of Dip G was further evaluated using a soft agar colony-formation assay (Figure 1d). Dip G pretreatment for 24 h reduced the colony number and colony size in the HL-60 cells compared with the untreated controls (Figure 1e). Similar results were also obtained in U937 cells following Dip G pretreatment (data not shown).

### Dip G induces AML cell differentiation

**Figure 2:**
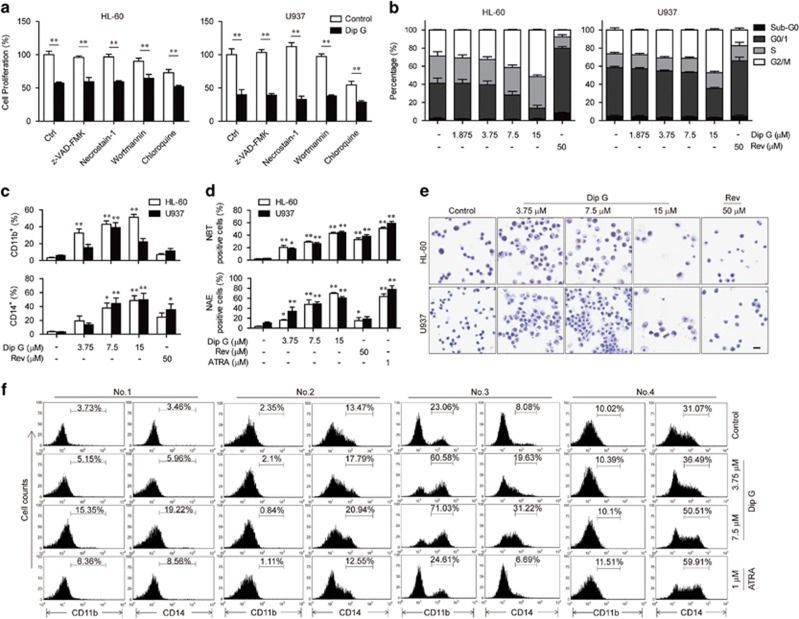
Differentiation-inducing activity of Dip G in AML cells. (**a**) HL-60 and U937 cells were treated for 72 h in the absence or presence of Dip G (7.5 *μ*M) with or without pretreatment with various inhibitors (20 *μ*M z-VAD-FMK, 30 *μ*M Necrostatin-1, 0.1 *μ*M Wortmanin or 20 *μ*M Chloroquine) for 2 h. The rate of cell proliferation was measured using an MTT assay. (**b**) The cells were treated in the presence of the indicated concentrations of Dip G or Rev (50 *μ*M) for 24 h, and cell cycle analysis was performed using flow cytometry. (**c**–**e**) The cells were treated for 72 h. (**c**) The percentage of cells expressing CD11b or CD14. *P<0.05, **P<0.01 versus the control group without any treatment. (**d**) The effect of Dip G on the reduction of nitroblue tetrazolium (NBT; upper panel) and *α*-naphthyl acetate esterase activity (lower panel). *P<0.05, **P<0.01 versus the control group without any treatment. (**e**) Representative Wright–Giemsa staining for morphological examination under a light microscope at magnification × 200. Scale bar, 200 *μ*m. (**f**) Primary AML cells were treated with various concentrations of Dip G or ATRA (1 *μ*M) for 72 h. CD11b or CD14 expression was detected using flow cytometry. Data are shown as the mean±S.E.M. of three independent experiments. *P<0.05, **P<0.01

To explore the antiproliferative activity of Dip G in AML cells, both HL-60 and U937 cells were pretreated with a pan-caspase inhibitor (z-VAD-FMK), a necroptosis inhibitor (Necrostatin-1) or an autophagy inhibitor (Wortmannin or Chloroquine) before treatment with 7.5 *μ*M of Dip G. These inhibitors failed to reverse the inhibitory effects of Dip G on cell proliferation (Figure 2a). Moreover, Dip G caused impaired cleavage of caspase 3, an apoptotic marker, even at 15 *μ*M compared with Rev in both AML cell lines (Supplementary Figure S1b). These results suggest that other mechanisms could possibly account for the Dip G-mediated inhibition other than the induction of caspase-dependent apoptosis, necroptosis or autophagic cell death.

Next, we examined the effects of Dip G on cell-cycle arrest. Dip G arrested the cell cycle at G2/M and decreased the G0/G1 phase in both cell lines in a dose-dependent manner (Figure 2b). In contrast, Rev treatment led to significant accumulation of cells in the G0/G1 phase, indicating the different mechanism of action between Dip G and Rev in AML cells.

Given that the progress of cancer cell differentiation is tightly coupled to cell growth arrest, we assessed AML cell differentiation using a standard assay for myeloid maturation. In the HL-60 cells, the levels of both myeloid (CD11b) and monocytic (CD14) markers were increased in a dose-dependent manner after incubation with Dip G for 72 h (Figure 2c). Similar results were obtained in U937 cells with the exception of the 15 *μ*M dose. Dip G also enhanced CD11b expression in resistant HL-60/Adr cells in a dose-dependent manner, whereas neither Rev nor ATRA had any effect (Supplementary Figure S2a). The production of oxidative bursts in HL-60 and U937 cells was increased by Dip G treatment (Figure 2d). In addition, these cells also showed an increase in *α*-naphthyl esterase activity, which was similar to the results of ATRA treatment. The differentiation-inducing capacity of Dip G was further confirmed using morphological analysis with Wright–Giemsa staining. Compared with the untreated cells, the Dip G-treated cells showed mature morphology with an increased cytoplasmic-to-nucleus ratio and chromatin condensation (Figure 2e). In keeping with the AML cell lines, Dip G increased the expression of both CD11b and CD14 in a dose-dependent manner in the cells from primary AML samples No.1 and No.3 (Figure 2f). The expression of CD14 only was increased in the cells from primary AML samples No.2 and No.4 following Dip G treatment. However, 1 *μ*M of ATRA had little or no effect on the expression of these markers in primary patient-derived AML cells. Primary AML cells treated with 7.5 *μ*M of Dip G also displayed morphological features of cell differentiation (Supplementary Figure S2b). These results implicate that Dip G might be equivalent to or better than ATRA in inducing myeloid differentiation of AML cells.

### STAT1 activation is involved in Dip G-induced AML differentiation

**Figure 3:**
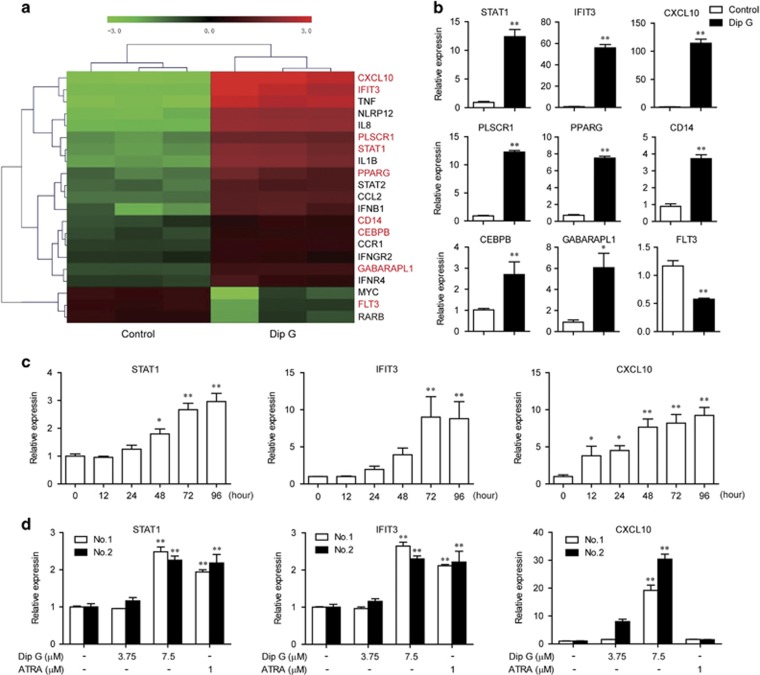
Induction of STAT1 activation by Dip G in AML cells. (**a**) Heat map of differentiation-related genes modulated by Dip G. HL-60 cells were treated for 24 h in the absence or presence of Dip G (15 *μ*M). The mRNA expression was detected using an Agilent Human Gene Expression Array. Eighteen upregulated genes and three downregulated genes were used to generate the heat map. Red gene symbols indicate myeloid differentiation markers. (**b**) The results of the microarray were verified using quantitative real-time reverse transcriptase-PCR (RT-PCR) analysis. The samples used for validation were the same samples as those that were used for the microarray. (**c**) HL-60 cells were treated with Dip G (7.5 *μ*M) for the indicated times and (**d**) primary AML cells were treated with various concentrations of Dip G or ATRA (1 *μ*M) for 24 h. The mRNA expression of STAT1, IFIT3 and C-X-C motif chemokine ligand 10 (CXCL10) was detected using quantitative real-time RT-PCR analysis. Glyceraldehyde 3-phosphate dehydrogenase was used as an internal control. Data are shown as the mean±S.E.M. of three independent experiments. *P<0.05, **P<0.01 versus the control group without any treatment

To gain insight into the mechanism underlying Dip G-induced AML differentiation, the gene expression profiles of HL-60 cells treated with Dip G were analyzed using the microarray. After 24 h of treatment, the Dip G-treated cells showed significant changes in the mRNA expression of 21 differentiation-related genes, including 18 upregulated genes and 3 downregulated genes (Figure 3a). Among the product of these genes, STAT1, the STAT1 target genes IFIT3 and CXCL10, phospholipid scramblase 1 (PLSCR1), peroxisome proliferator activated receptor gamma (PPARG), CD14, C/EBPB, GABA (A) receptor-associated protein like 1 and FLT3 are known myeloid differentiation markers. The microarray data were validated using qPCR (Figure 3b and Supplementary Figure S3a). Of the multiple canonical pathways, the Jak-STAT and MAPK signaling pathways were overrepresented (Supplementary Figure S3b). Owing to their conspicuous increase in expression after Dip G treatment, we focused on STAT1 and its direct target genes. A time-dependent increase was also observed in the expression of these genes (Figure 3c). Consistently, 7.5 *μ*M of Dip G also led to a significant increase in the mRNA expression of STAT1, IFIT3 and CXCL10 in primary AML cells (Figure 3d).

### Dip G promotes the nuclear translocation of p-STAT1 (Ser727)

**Figure 4:**
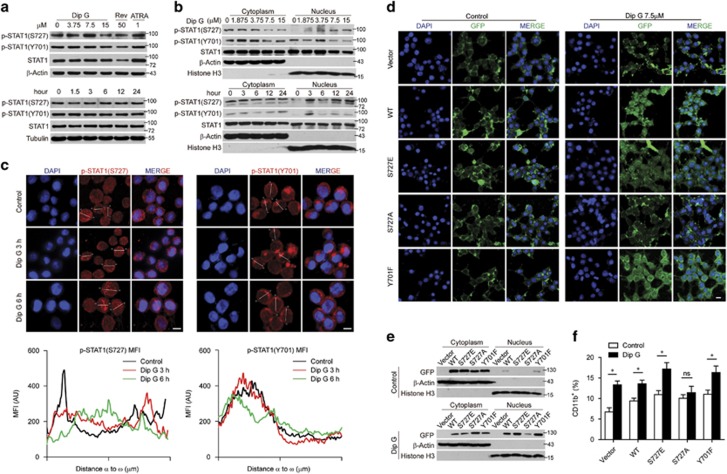
Nuclear translocation of phospho-STAT1 (Ser727) promoted by Dip G. (**a** and **b**). HL-60 cells were treated with the indicated compounds for 24 h or with Dip G (7.5 *μ*M) for the indicated times. (**a**) The protein levels of p-STAT1 (S727 and Y701) and STAT1 were analyzed in the whole-cell lysate using western blotting. *β*-Actin or tubulin was used as a loading control. (**b**) Western blotting analysis for the indicated protein levels in the cytoplasm and nucleus. *β*-Actin or histone 3 was used as a loading control. (**c**) HL-60 cells were treated with Dip G (7.5 *μ*M) for 3 or 6 h. The subcellular location of p-STAT1 (S727) and p-STAT1 (Y701) was detected using confocal microscopy. The nuclei are stained with the DNA-binding dye DAPI (4,6-diamidino-2-phenylindole; blue). Scale bar, 10 *μ*m. The lower panel represents the mean fluorescence intensity (MFI), which is presented in arbitrary units (AU), and the distance from *α* to *ω* in the images. (**d** and **e**). STAT1-WT or STAT1 mutants were overexpressed in HeLa cells. (**d**) Twenty-four hours after transfection, the resulting cells were treated with Dip G (7.5 *μ*M) for 6 h. Representative photomicrograghic images show the translocation of exogenous STAT1. Scale bar, 10 *μ*m. (**e**) The resulting cells were treated with Dip G (7.5 *μ*M) for an additional 6 h. The protein levels of exogenous STAT1 in the cytoplasm and nucleus were analyzed using western blotting. (**f**) STAT1-WT or STAT1 mutants were overexpressed in HL-60 cells using electroporation. The resulting cells were treated with Dip G (7.5 *μ*M) for 72 h. CD11b expression on gated green-fluorescent protein (GFP)-positive cells was detected using flow cytometry. Data are shown as the mean±S.E.M. of three independent experiments. *P<0.05; NS, *P*>0.05

Given that the phosphorylation of STAT1 at Tyr701 and Ser727 is critical for STAT1 activation,^20, 25^ we determined whether Dip G-induced differentiation is dependent on increased STAT1 phosphorylation. Contrary to our expectations, although Dip G is capable of increasing STAT1 mRNA expression in AML cells, there were no significant changes in total STAT1 and p-STAT1 (Ser727) protein levels in the whole-cell lysates of Dip G-treated HL-60 cells except for a slight decrease in the protein levels of p-STAT1 (Tyr701) (Figure 4a). In contrast, ATRA upregulated the protein levels of STAT1 and p-STAT1 (Ser727 and Tyr 701). Surprisingly, p-STAT1 (Ser727) nuclear accumulation was observed following Dip G treatment (Figure 4b). P-STAT1 (Ser727) translocated from the cytoplasm to the nucleus in a dose- and time-dependent manner. The nuclear translocation of p-STAT1 (Ser727) was further confirmed using confocal microscopy and *z*-stack acquisition (Figure 4c). P-STAT1 (Ser727) was mainly located in the cytoplasm of undifferentiated HL-60 cells and translocated to the nucleus after 6 h of Dip G treatment, whereas Dip G did not promote p-STAT1 (Tyr701) nuclear accumulation.

Next, we detected exogenous STAT1 nuclear translocation. HeLa cells were transiently transfected with EGFP-STAT1-WT plasmids and EGFP-STAT1 mutants (S727E, S727A, Y701F). When the resulting cells were treated with 7.5 *μ*M of Dip G for 6 h, STAT1-WT translocated from the cytoplasm to the nucleus, which was consistent with the endogenous STAT1 translocation pattern assessed using confocal microscopy (Figure 4d). Similar patterns of nuclear translocation of STAT1-WT were observed following S727E mutations, which mimic the phosphorylation of Ser727, or phosphorylation-deficient Y701F mutations. In sharp contrast, mutation of Ser727 to alanine greatly suppressed Dip G-induced STAT1 nuclear translocation. Similar results were observed using western blotting (Figure 4e). We hypothesized that the phosphorylation of STAT1 at Ser727, but not at Tyr701, might be necessary for Dip G-induced differentiation. Electroporation was used to overexpress STAT1-WT or its mutants in HL-60 cells. STAT1 (S727A) overexpression abolished the Dip G-induced increase in CD11b expression, whereas S727E or Y701F mutations had no effects compared with STAT1-WT (Figure 4f). Taken together, our data indicate that p-STAT1 (Ser727) has a critical role in Dip G-mediated STAT1 nuclear translation and subsequent AML differentiation.

### ERK phosphorylation is required for nuclear translocation of p-STAT1 (Ser727)

**Figure 5:**
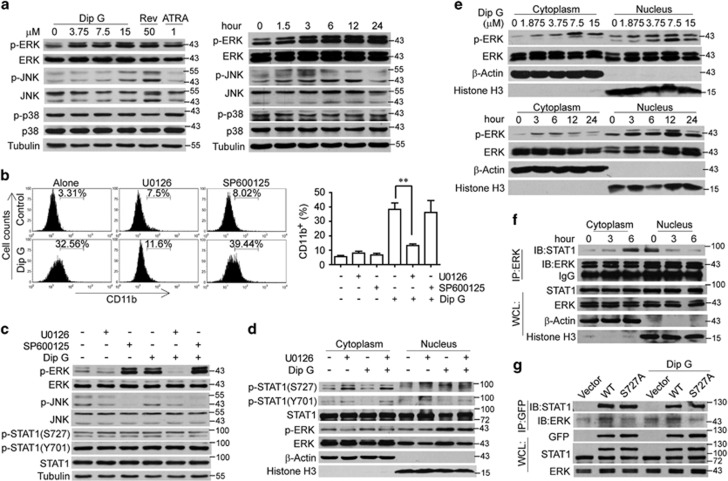
Nuclear translocation of p-STAT1 (Ser727) driven by extracellular signal–regulated kinase (ERK) activation. (**a**) HL-60 cells were treated with the indicated compounds for 24 h or with Dip G (7.5 *μ*M) for the indicated times. Mitogen-activated protein kinase signaling in the whole-cell lysates were analyzed using western blotting. Tubulin was used as a loading control. (**b**) HL-60 cells were treated with Dip G (7.5 *μ*M) in the absence or presence of U0126 (1 *μ*M) or SP610025 (10 *μ*M) for 72 h. CD11b expression was detected using flow cytometry. Right panel: the percentage of cells expressing CD11b. Data are shown as the mean±S.E.M. of three independent experiments. *P<0.05. (**c** and **d**). HL-60 cells were treated with Dip G (7.5 *μ*M) in the absence or presence of U0126 (1 *μ*M) or SP610025 (10 *μ*M) for 24 h. (**c**) The protein levels of p-ERK, ERK, phospho-c-Jun N-terminal kinase (p-JNK), JNK, p-STAT1 (S727 and Y701) and STAT1 in the whole-cell lysates were analyzed using western blotting. Tubulin was used as a loading control. (**d**) Western blotting analysis for the indicated protein levels in the cytoplasm and nucleus. *β*-Actin or histone 3 was used as a loading control. (**e**) HL-60 cells were treated with various concentrations of Dip G for 24 h or with Dip G (7.5 *μ*M) for the indicated times. The protein levels of p-ERK and ERK in the cytoplasm and nucleus were analyzed using western blotting. (**f**) HL-60 cells were treated with Dip G (7.5 *μ*M) for the indicated times. The interaction between STAT1 and ERK was measured using an immunoprecipitation assay. (**g**) STAT1-WT or STAT1 (S727A) mutants were overexpressed in HeLa cells. The resulting cells were treated with Dip G (7.5 *μ*M) for an additional 6 h. The interaction between STAT1 and ERK was measured

Given that the MAPK cascade mediates the phosphorylation of STAT1 (Ser727) in response to various stimuli,^21, 22^ we detected the effect of Dip G on MAPKs in HL-60 cells. Dip G treatment led to a dose- and time-dependent increase in the phosphorylation of ERK with a minimal effect on the phosphorylation of JNK and p38 (Figure 5a). The level of p-ERK plateaued after 6 h of Dip G treatment. To determine whether ERK activation is required for the induction of AML differentiation by Dip G, HL-60 cells were treated with Dip G in the presence of the ERK inhibitor (U0126) or JNK inhibitor (SP600125) and CD11b expression was assessed. U0126 significantly relieved the Dip G-induced increase in CD11b expression, whereas little change was detected with SP600125 (Figure 5b). As expected, the activation of ERK and JNK was successfully inhibited by their inhibitors (Figure 5c). However, neither of the inhibitors affected the phosphorylation of STAT1 at Ser727 and Tyr701 in the whole-cell lysates. These results suggest that ERK-medicated STAT1 phosphorylation might not be sufficient to account for Dip G-induced AML differentiation.

To investigate whether there is a link between ERK activation and Dip G-mediated STAT1 nuclear translation, we determined the effect of ERK inactivation on STAT1 nuclear translocation in Dip G-treated HL-60 cells. Compared with the untreated controls, Dip G markedly promoted the nuclear translocation of p-STAT1 (Ser727), which was blocked by U0126 (Figure 5d). A reduction of nuclear p-ERK, but not p-STAT1 (Tyr701), was also observed in the cell treated with both Dip G and U0126. In addition, Dip G caused the nuclear and cytoplasmic accumulation of p-ERK in a dose- and time-dependent manner (Figure 5e), which suggested that p-ERK could be related to the nuclear trafficking of p-STAT1 (Ser727).

Next, we determined the effect of Dip G on the interaction between ERK and STAT1. Immunoprecipitation of ERK from the cytoplasm or nuclear protein fractions of the treated HL-60 cells followed by western blotting for STAT1 demonstrated that ERK and STAT1 bind to each other (Figure 5f). More importantly, this interaction gradually increased in the cytoplasm and decreased in the nucleus over time, which indicates that Dip G might facilitate the formation of a complex containing ERK and STAT1 in the cytoplasm and induce its disaggregation in the nucleus. When the interaction between ERK and exogenous STAT1 was evaluated using immunoprecipitation, ERK was bound to STAT1-WT, but not to S727A mutant (Figure 5g). Therefore, nuclear translocation of the p-STAT1 (Ser727)/p-ERK complex may be crucial for the differentiation-inducing capacity of Dip G.

### Dip G suppresses HL-60 cell growth *in vivo* by inducing differentiation

**Figure 6:**
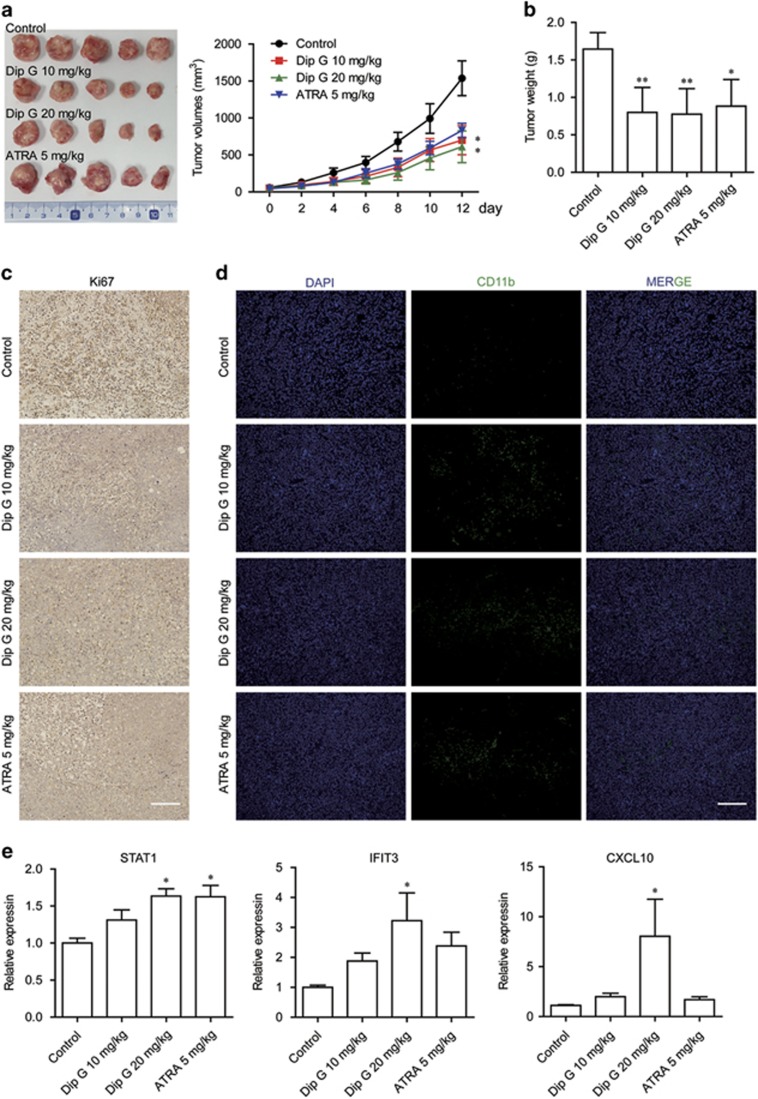
*In vivo* therapeutic efficacy of Dip G. HL-60 cells were injected subcutaneously into the right flank of NOD/SCID mice. Two weeks later, tumor-bearing mice were distributed into four groups and treated with various doses of Dip G or ATRA intraperitoneally daily for an additional 12 days. (**a**) Tumor volumes were monitored and recorded every 2 days (*n*=8–10 mice per group). Left panel: Representative images of the tumors. (**b**) Tumors excised on day 13 were weighed. Tumors excised on day 13 were stained with an antibody specific for (**c**) Ki67 and (**d**) CD11b. Scale bar, 100 *μ*m. (**e**) The mRNA expression of STAT1, IFIT3 and C-X-C motif chemokine ligand 10 (CXCL10) was detected in tumors excised on day 13 using quantitative real-time reverse transcriptase-CR analysis. Glyceraldehyde 3-phosphate dehydrogenase was used as an internal control. Data are shown as the mean±S.E.M. of three independent experiments. *P<0.05, **P<0.01 versus the control group with phosphate-buffered saline treatment. DAPI, 4,6-diamidino-2-phenylindole

To evaluate the therapeutic efficacy of Dip G, we performed xenograft experiments in SCID mice that received transplanted HL-60 cells subcutaneously. Treatment of animals with two doses of Dip G (10 and 20 mg/kg) dramatically inhibited the growth of HL-60 cells *in vivo* (Figure 6a). In contrast, no profound change in tumor volume was observed following administration of a suboptimal dose of ATRA (5 mg/kg). When the tumors were removed on day 13, the average tumor weight was approximately two-fold less in the mice treated with either 10 or 20 mg/kg of Dip G compared with the vehicle controls (Figure 6b). Dip G did not cause weight loss in the animals or decrease the liver and spleen weights (Supplementary Figure S4a), which indicates that Dip G has less adverse effects. Positive immunostaining for Ki67 and CD11b revealed that the HL-60 tumors from the Dip G- or ATRA-treated mice had a decrease in cell proliferation and a substantial increase in CD11b-positive cells (Figures 6c and d). Terminal deoxynucleotidyl transferase dUTP nick end labeling (TUNEL) assay revealed very low levels of apoptosis in the tumors from the treated group (Supplementary Figure S4b). In contrast, increased expression of p-ERK was detected in the treated tumors compared with the vehicle-treated controls (Supplementary Figure S4c). Moreover, Dip G treatment enhanced the mRNA expression of STAT1, IFIT3 and CXCL1 in the tumors, which was statistically significant at the higher dose of Dip G (Figure 6e). In contrast, there was no profound increase in IFIT3 and CXCL1 expression following ATRA administration. These results suggest that Dip G can inhibit the growth of human AML cells *in vivo* by inducing STAT1-associated differentiation.

## Discussion

Although differentiation therapy using ATRA has significantly improved the prognosis for patients with APL, the other approximately 90% of AML types fail to respond to pharmacological doses of ATRA.^6, 27^ Therefore, many efforts have been made to find alternative differentiating agents. Here we demonstrate that Dip G exhibits potential antiproliferative activity by inducing AML cell differentiation both *in vitro* and *in vivo*. This compound triggered ERK activation, which caused phosphorylation of STAT1 at Ser727 and selectively enhanced the interaction of p-STAT1 (Ser727) and p-ERK leading to their nuclear translocation, which, in turn, is necessary for further differentiation of the AML cells.

Leukemia cells are often inhibited in their hematopoietic differentiation by gene expression abnormalities, such as the transcriptional repression of differentiation-related genes.^28^ HL-60 gene expression profiling, using a microarray, demonstrated that Dip G modulated the mRNA expression of a number of genes. We focused on some of the differentiation-related genes that include myeloid differentiation markers (e.g., STAT1, IFIT3, CXCL10, PLSCR1 and PPARG), cytokines and chemokines (e.g., IL-1*β*, TNF and CCR1) and transcription factors (e.g., STAT2 and c-Myc). In particular, the expression of STAT1 and its direct target genes IFIT3 and CXCL10 were markedly upregulated and prolonged by Dip G. It has been reported that STAT1 induction and activation have an important role in myeloid differentiation mainly through the regulation of a subset of IFN-*γ*-inducible genes, such as the upregulation of CXCL10 and the downregulation of c-Myc.^25, 29, 30^

Although ATRA treatment induces STAT1 both at the mRNA and protein levels in AML cells,^16, 18^ Dip G had no influence on the protein expression of total STAT1. In addition, ATRA augmented the levels of p-STAT1 (Ser727 and Tyr 701) in the nuclear and cytoplasmic protein fractions, whereas only the levels of p-STAT1 (Ser727) were increased in the nuclear fraction compared with the cytoplasmic fraction in Dip G-treated HL-60 cells (Supplementary Figure S5), suggesting that, although they both induce STAT1 activation, the mechanism of action of Dip G is different from ATRA. The results obtained from the mutation experiments highlight an important point: Dip G-induced STAT1 nuclear translocation is independent of the tyrosine phosphorylation of STAT1 on Tyr701. Y701F mutation did not affect p-STAT1 (Ser727) trafficking into the nucleus and subsequent AML differentiation. In contrast, the phosphorylation of STAT1 (Ser727) was necessary. Consistently, previous studies demonstrated that STAT1 tyrosine phosphorylation is not required for STAT1 serine phosphorylation and that serine-phosphorylated STAT1 can be localized in the cytoplasm where STAT proteins associate with other cytoplasmic proteins.^31, 32, 33^ We found that Dip G enhanced the interaction of p-STAT1 (Ser727) and p-ERK in the cytoplasm, further promoting their nuclear translocation and transactivation. Mutation of Ser727 to alanine disrupted the interaction between ERK and STAT1. These data, for the first time, suggest that a small molecule can selectively drive p-STAT1(Ser727) nuclear translocation independent of the phosphorylation of STAT1 (Tyr701). Ser727 phosphorylation of STAT1 is necessary for maximal transcriptional activity of STAT1 and is important for AML differentiation.^25, 29, 30^ On the other hand, increased phosphorylation of STAT1 (Tyr701) can promote the proliferation of AML cells.^34^ And increased tyrosine phosphorylation of STAT1 might interfere with differentiation of AML cells.^11^

ERK activation was necessary for Dip G-induced AML differentiation. Dip G treatment led to a substantial increase in ERK phosphorylation in HL-60 and primary AML cells (Figure 5a and Supplementary Figure S6a). Pharmacological inhibition of ERK with U0126 blocked the nuclear translocation of p-STAT1 (Ser727) and subsequent AML differentiation. However, the mechanism by which Dip G drives the nuclear translocation of the p-STAT1 (Ser727)/p-ERK complex needs further investigation. ERK activation is positively regulated by various cellular mediators, such as Ras, ROS and PKC.^35, 36, 37^ Dip G had no effect on the activation of Src homology 2 domain-containing tyrosine phosphatase 2 (Supplementary Figure S6b), a positive regulator of the Ras/Raf/MEK/ERK pathway.^38^ In addition, Dip G had no effect on ROS accumulation in the treated cells and the PKC inhibitor Gö 6983 failed to alleviate the Dip G-induced increase in CD11b expression (Supplementary Figures S6c and d), suggesting that the Dip G-induced ERK activation is not related to Ras activation, ROS induction or PKC signaling. Further investigation of the mechanism of Dip-G-induced ERK activation is ongoing in our laboratory.

ATRA acts mainly through a family of nuclear receptors, including RARs, which activate the transcription of retinoid acid response element (RARE)-regulated target genes.^39^ A RARE domain reportedly exists in the STAT1 promoter.^15^ Although other compounds, such as 1,25-dihydroxyvitamin D_3_, are capable of inducing AML cell differentiation through a RAR*α*-independent mechanism, side effects limit their clinical use.^28^ Dip G shows a unique biological action that modulates ERK activation and nuclear translocation of STAT1 (Ser727), which is distinct from both its analog Rev and ATRA. This novel mechanism of action may be clinically important for differentiation therapy in AML, particularly for non-APL AML therapy.

## Materials and methods

### Chemicals

Dip G (>98% purity) was isolated as previously described.^9^ Rev, ATRA, chloroquine, U0126, SP600125, phorbol myristate acetate (PMA) and Gö 6983 were purchased from Sigma-Aldrich (St. Louis, MO, USA). z-VAD-FMK, Necrostatin-1 and wortmannin were from Selleck (Houston, TX, USA).

### Cell culture and transfection

HL-60, HL-60/Adr (Adriamycin resistant) and U937 (Institute of Hematology, Chinese Academy of Medical Sciences, Tianjin, China) cells were cultured in RPMI 1640. HeLa cells (American Type Culture Collection, Rockville, MD, USA) were grown in DMEM. Primary leukemic cells from AML patients without prior therapy (the First Affiliated Hospital of Nanjing Medical University, Nanjing, China) were collected using lymphocyte-monocyte separation medium (KeyGen BioTECH, Nanjing, China), after obtaining informed consent, in agreement with the hospital’s Institutional Review Board and in accordance with the Declaration of Helsinki. The procedures were approved by the appropriate ethics committees.

EGFP-STAT1-WT plasmids, EGFP-STAT1 mutants (S727A, S727E, Y701F) and EGFP-pcDNA3.1 vectors were obtained from Addgene (Cambridge, MA, USA). HL-60 cells were electrotransfected with plasmids using Neon Transfection System MPK5000 (Invitrogen, Carlsbad, CA, USA). HeLa cells were transfected using Lipofectamine 3000 (Invitrogen).

### Cell viability and proliferation assay

Cells (5 × 10^3^ cells/well) were seeded in 96-well plates and treated with the indicated compounds. The number of cells was assessed using the Trypan Blue dye exclusion method with manual cell counting using a hemocytometer (XB.K.25, QIUJING, Shanghai, China). The rate of cell proliferation was detected using BrdU incorporation analysis and MTT assay, respectively.^40^ BrdU assay was carried out using BrdU Cell Proliferation Assay Kit (Cell Signaling Technology, Beverly, MA, USA) according to the manufacturer’s instructions. Soft agar colony-formation assays were carried out after the cells were pretreated with Dip G or Rev for 24 h and then cultured in six-well plates for another 20 days.^41^ The plates were photographed using a digital camera (Olympus, Tokyo, Japan). The number and size of colonies were detected at × 40 magnification using a Nikon inverted microscope (Tokyo, Japan).

### Cell cycle and differentiation analysis

The cell cycle was analyzed by measuring propidium iodide staining using a FACSCalibur flow cytometer (Becton Dickinson, San Jose, CA, USA).^42^ The percentage of cells in each phase of the cell cycle was quantified with the ModFit software (Becton Dickinson). The expression of cell surface differentiation markers (CD14 and CD11b) was analyzed using a FACSCalibur flow cytometer. The treated cells were tested using an *α*-Naphthyl Acetate Esterase Assay Kit (Sigma-Aldrich). The percentage of cells capable of reducing nitroblue tetrazolium was measured as described previously.^43^ Morphological assessment of differentiation was evaluated using a Wright–Giemsa Staining Kit (Jiancheng Bioengineering Institute, Nanjing, China).

### Microarray assay

The Agilent Human Gene Expression Microarray Kit (Design ID: 3039494, Agilent Technologies, Santa Clara, CA, USA) was used for gene profiling. The sample labeling and microarray hybridization were performed based on the manufacturer’s standard protocols. Feature Extraction software (version 10.7.1.1, Agilent Technologies) was used to analyze the array images to obtain the raw data. All microarray data are available at the Gene Expression Omnibus at NCBI under accession number GSE84652.

### Quantitative real-time RT-PCR analysis

RNA samples were reverse transcribed to cDNA and subjected to quantitative PCR, which was performed using the BioRad CFX96 Touch Real-Time PCR Detection System (Bio-Rad, Hercules, CA, USA) and iQSYBR Green Supermix (Bio-Rad), and threshold cycle numbers were obtained using the BioRad CFX Manager software version 5.0. The conditions for amplification were 1 cycle at 95 °C for 2 min followed by 40 cycles at 95 °C for 15 s, 60 °C for 30 s and 95 °C for 10 s. The primer sequences used in this study are listed in the Supplementary Table S1.

### Western blotting and immunoprecipitation

Western blot analysis and immunoprecipitation were performed as previously described.^44^ Briefly, the cells were collected and lysed in lysis buffer containing protease inhibitor (protease inhibitor cocktail, Pierce, Rockford, IL, USA). The proteins were fractionated by SDS-PAGE and electrotransferred to polyvinylidene fluoride membranes (Millipore Corp., Bedford, MA, USA). Primary antibodies against p-STAT1 (S727), p-STAT1 (Y701), STAT1, p-ERK, ERK and Histone 3 were purchased from Cell Signaling Technology. In addition, we also used antibodies against p-JNK and JNK (Abcam, Cambridge, MA, USA), p-p38 and p38 (Epitomics, Burlingame, CA, USA), GFP, tubulin and *β*-actin (Abmart, Shanghai, China). NE-PER Nuclear and Cytoplasmic Extraction Reagents were obtained from Thermo Fisher Scientific (Rockford, IL, USA). Western blot detection was performed using a ChemiDoc XRS+chemiluminescent substrate system (Bio-Rad).

For immunoprecipitation, the cell lysate was precleared by adding 1.0 *μ*g of the appropriate control IgG together with 50 *μ*l of resuspended volume of Protein A/G PLUS-Agarose and incubated at 4 °C for 30 min. The supernatant was incubated with primary antibody overnight at 4 °C. A resuspended volume of 50 *μ*l Protein A/G was added to the complex and incubated on a rotating device at 4 °C for 4 h. The immunoprecipitates were collected by centrifugation at 1000 r.c.f. for 5 min, and the pellets were washed four times with lysis buffer and resuspended in 40 *μ*l 1 × electrophoresis sample buffer. Boilled samples were analyzed by western blotting.

### Immunofluorescence analysis

Subcellular localization of STAT1 was analyzed using immunofluorescent microscopy according to previously described methods.^44^ Briefly, cells on coverslips were washed twice with PBS and fixed in Fixation buffer at 4 °C for 15 min. Permeabilization of the cells was performed by incubation with 1 × PhosflowTM Perm Buffer on ice for 30 min. The cells were blocking with 3% BSA for 1 h and rinsed three times with PBS and then incubated with the primary antibody against p-STAT1(S727) or p-STAT1(Y701) at 4 °C overnight. For immunofluorescence detection of STAT1, some cells were incubated with a secondary antibody (Alexa Fluor-594, Invitrogen) for 2 h. The nucleus was stained with DAPI for 2 min. After staining, the cells were rinsed four times with PBS and prepared for microscopic analysis. Images were acquired using fluorescence microscopy.

### *In vivo* experiments

Cultured HL-60 cells (1 × 10^7^ cells in 0.1 ml PBS) were injected into the right flank of NOD/SCID mice (Shanghai Laboratory Animal Center, Shanghai, China). Two weeks after the injection, the mice bearing tumors (an average size of 50 mm^3^) were distributed into four groups (*n*=8–10 mice per group). Dip G (10 or 20 mg/kg) or ATRA (5 mg/kg, 1:99, v/v DMSO: PBS) was administered daily for 12 days intraperitoneally. Tumor volumes were measured every 2 days and calculated using the following formula: 0.5236 × L1 × (L2)^2^, where L1 and L2 are the long and short diameters of the tumor mass, respectively. The tumor tissue, liver and spleen were excised and weighed on day 13.

### Immunohistochemistry

Tumor tissues were embedded in paraffin. For immunohistochemical analyses, deparaffinized sections were subjected to antigen retrieval in 0.01 M citrate buffer solution. After blocking of endogenous peroxidase activity in 3% H_2_O_2_, the sections were incubated with anti-Ki67 mAb (Cell Signal Technology), anti-CD11b or anti-p ERK mAb (BD Pharmingen, San Diego, CA, USA) overnight at 4 °C. Then the sections were rinsed and visualized by immunoperoxidase staining with the Real Envision Detection Kit (GeneTech, Shanghai, China) according to the manufacturer’s instructions or detected by Immunofluorescence analysis. TUNEL assay was performed using the *In Situ* Cell Death Detection Kit (Roche, Basel, Switzerland).

### Statistical analysis

Data are expressed as the mean±S.E.M. Statistical analysis was performed using Student’s *t*-test and one-way ANOVA. All statistical analyses were conducted using the SPSS version 10.0 statistical software (SPSS, Chicago, IL, USA). A *P*-value of <0.05 was considered statistically significant.
