# Supplementary material for: Diptoindonesin G promotes ERK-mediated nuclear translocation of p-STAT1 (Ser727) and cell differentiation in AML cells
Source: Cell Death Dis. 2017 May 4;8(5):e2765–. doi: 10.1038/cddis.2017.159 (PMC5520695; doi:10.1038/cddis.2017.159)
Supplement: Supplementary Information [file cddis2017159x1.pdf]

Diptoindonesin G promotes ERK-mediated nuclear translocation of p-STAT1 (Ser727) and cell differentiation in AML cells

Jian Gao[1], Minmin Fan[1], Gang Xiang[1], Jujuan Wang[2], Xiong Zhang[1], Wenjie Guo[1], Xuefeng Wu[1], Yang Sun[1], Yanhong Gu[3], Huiming Ge[1], Renxiang Tan[1,4], Hongxia Qiu[*, 2],Yan Shen[*, 1], Qiang Xu[*, 1,4]

Table S1. Primer sequences for RT-PCR

Primer	Forward (5′→3′)	Reverse (5′→3′)
STAT1	GGTCTCCTCTGACTTCAACA	AGCCAAATTCGTTGTCATAC
IFIT3	AACTACGCCTGGGTCTACTATCACT	ACACCTTCGCCCTTTCATTTC
CXCL10	GAATCGAAGGCCATCAAGAA	GCTCCCCTCTGGTTTTAAGG
PLSCR1	CTGACTTCTGAGAAGGTTGC	GAATGCTGTCGGTGGATACTG
PPARG	TCAGGTTTGGGCGGATGC	TCAGCGGGAAGGACTTTATGTATG
CD14	ACGCCAGAACCTTGTGAGC	GCATGGATCTCCACCTCTACTG
CEBPB	CTTCAGCCCGTACCTGGAG	GGAGAGGAAGTCGTGGTGC
GABARAPL1	TTTGGTGCCCCTTATCTCAC	GGCCATCATGTAGCATTCCTT
FLT3	AGGGACAGTGTACGAAGCTG	GCTGTGCTTAAAGACCCAGAG
RARB	ATGCTGGATTTGGTCCTCTG	TGCACCTTTAGCACTGATGC
IL-8	GCAGAGGGTTGTGGAGAAG	GGCATCTTCACTGATTCTTGG
IL-1β	CCTGTCCTGCGTGTTGAAAG	TGCTTGAGAGGTGCTGATG
TNF	TGGCCCAGGCAGTCAGA	GGTTTGCTACAACATGGGCTACA
CCR1	CCTGCTGACGATTGACAGGTA	TTGGAAAAGTATAAGCCTGGCAT
CCL2	AGTCTCTGCCGCCCTTCT	GTGACTGGGGCATTGATTG
NLRP12	CATGATGCTGCTTTGCGA	TCCATCCCAAATAACCAGAGG
GAPDH	GGTCTCCTCTGACTTCAACA	AGCCAAATTCGTTGTCATAC

Supplementary Figures

Supplementary Figure S1

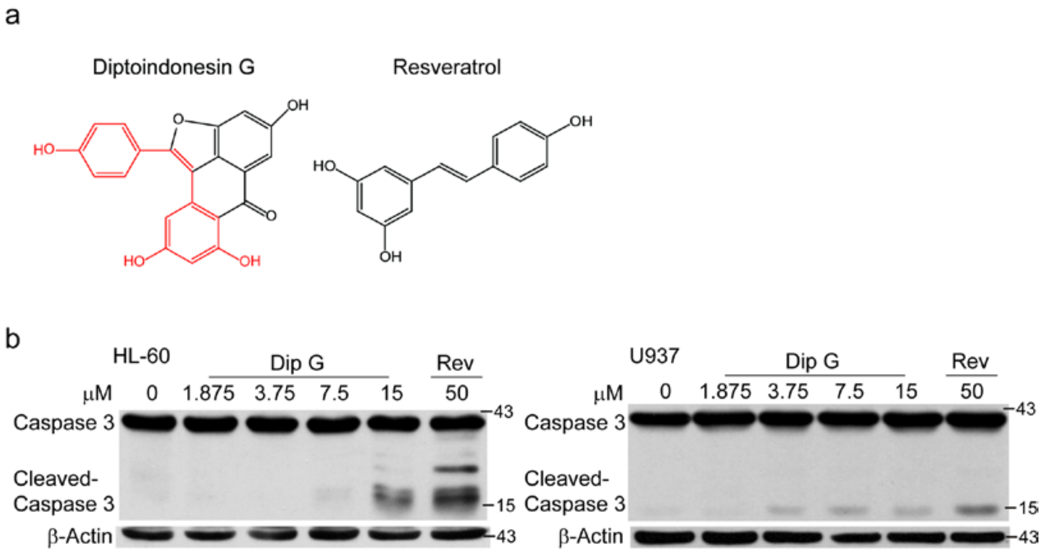

Figure S1. Effect of Dip G on apoptosis in AML cells. (a) The chemical structures of diptoindonesin G (Dip G) and resveratrol (Rev). (b) The cells were treated with Dip G or Rev for 24 h. Western blot analysis for caspase 3.

Supplementary Figure S2

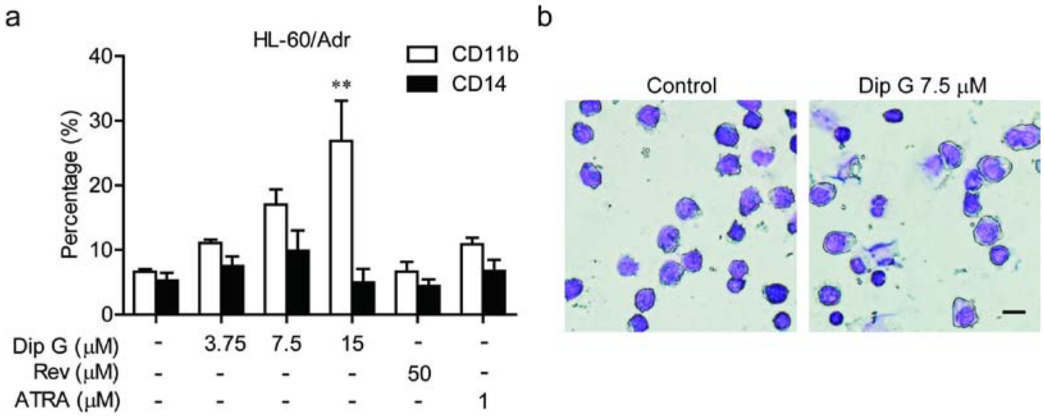

Figure S2. Effects of Dip G on differentiation in AML cells. (a) CD11b and CD14 expression in HL-60/Adr cells after 72 h of treatment. Data are shown as the mean ± SEM of three independent experiments. ***P*<0.01 versus the

control group without any treatment. (b) Representative Wright-Giemsa staining for morphological examination of primary AML cells treated with Dip G (7.5 μM) for 72 h. Scale bar, 200 μm.

Supplementary Figure S3

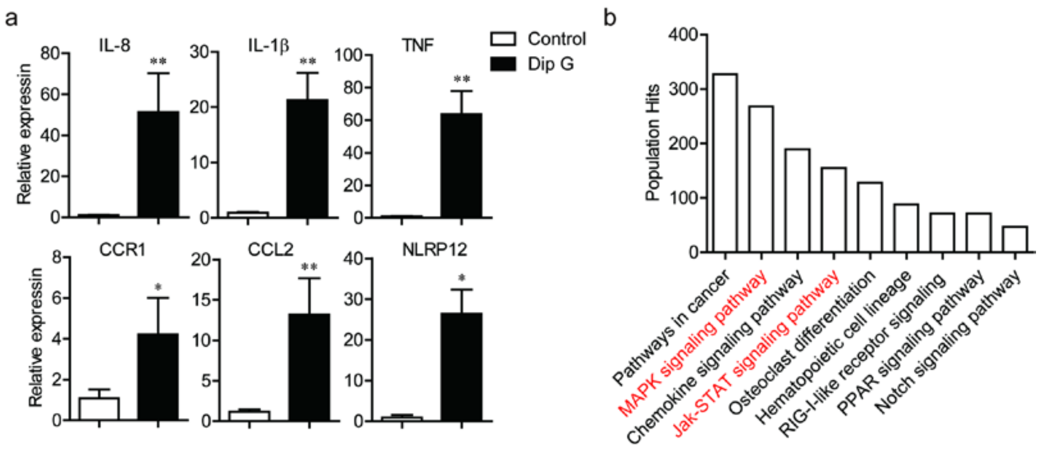

Figure S3. Regulation of gene profiling by Dip G. (a) Quantitative real-time RT-PCR analysis for the results of the microarray as described in Figure 3. GAPDH was used as an internal control. Data are shown as the mean ± SEM of three independent experiments. **P*<0.05, ***P*<0.01 versus the control group without any treatment. (b) Analysis of the canonical pathways enriched in the genes regulated by Dip G.

Supplementary Figure S4

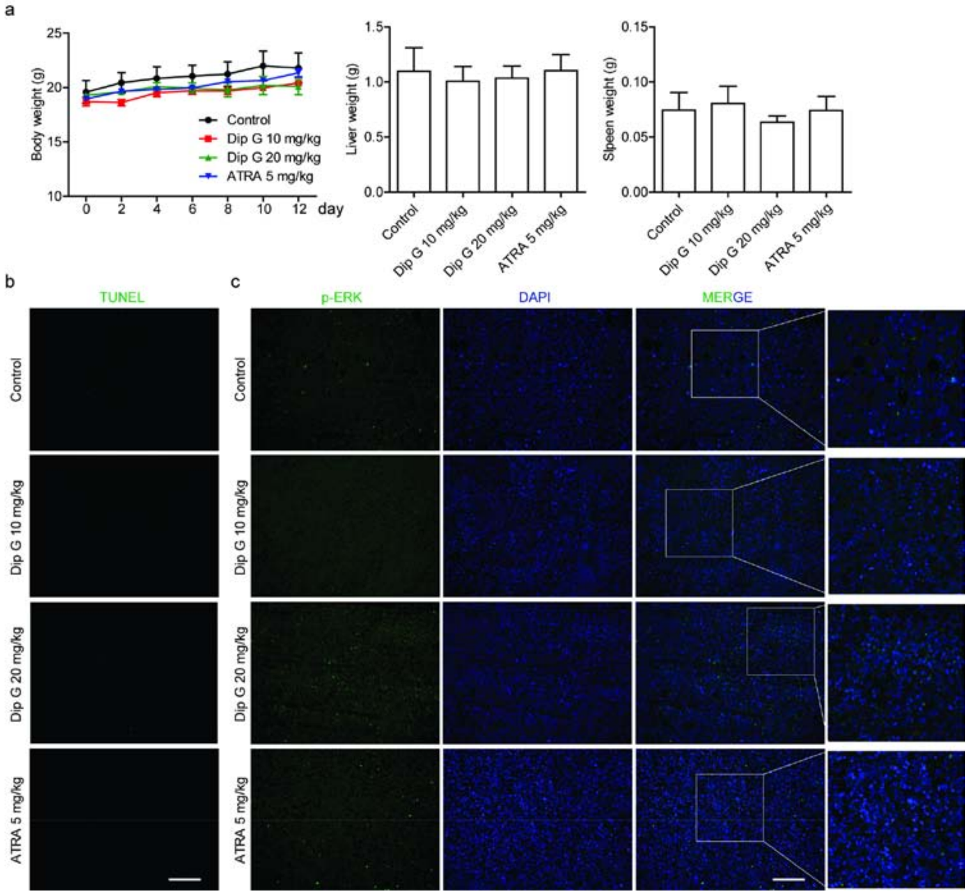

Figure S4. Effects of Dip G on body, liver and spleen weight in mice and cell differentiation in tumor tissues. Mice were treated as described in Figure 6. (a) left panel: body weight curves, middle panel: liver weight, right panel: spleen weight. Data are shown as the mean ± SEM of three independent experiments. Tumors were excised on day 13. (b) TUNEL staining. (c) Positive immunostaining for p-ERK in the tumor tissues. Scale bar, 100 μm.

Supplementary Figure S5

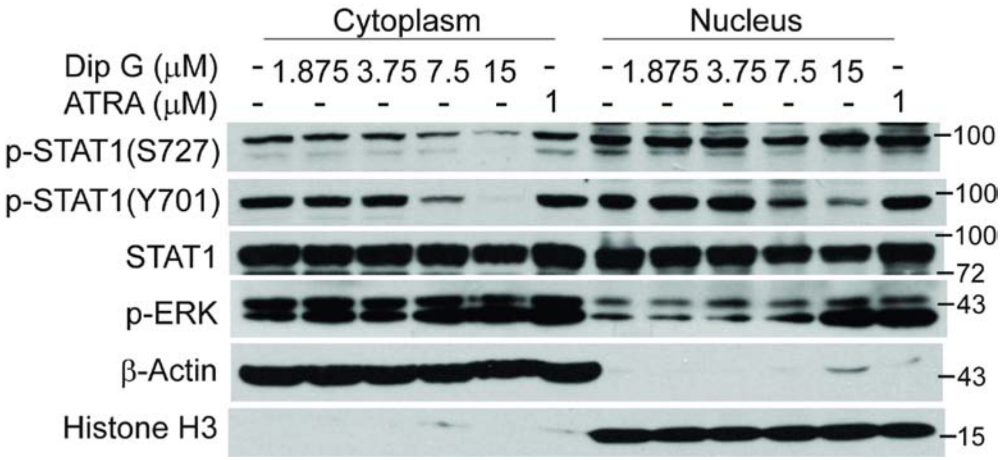

Figure S5. Effects of Dip G on nuclear translocation of STAT1 in AML cells. HL-60 cells were treated with Dip G or ATAR for 24 h. Western blotting analysis for the levels of the indicated proteins in the cytoplasm and nucleus.

Supplementary Figure S6

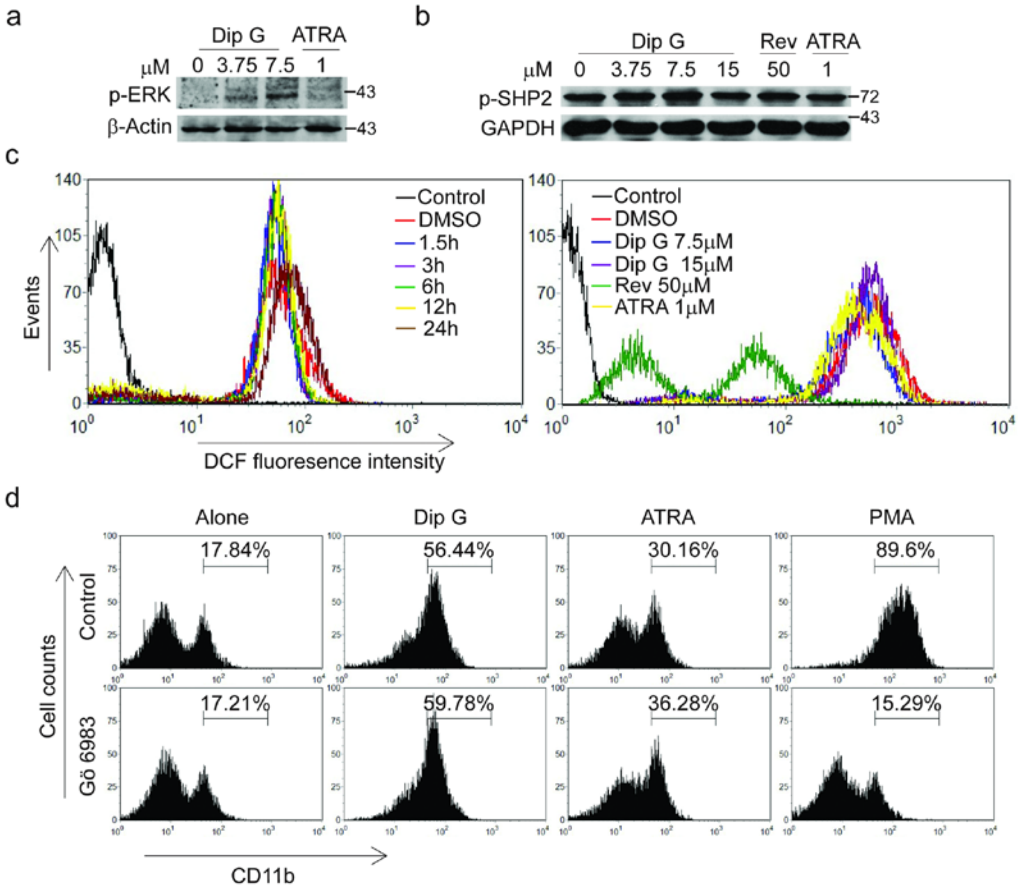

Figure S6. Effects of Dip G on phosphorylation of ERK and SHP-2, ROS production and PKC activation. (a) The protein levels of p-ERK in primary AML cells treated with Dip G for 24 h. (b) The protein levels of p-SHP2 in HL-60 cells treated with Dip G, ATAR or Rev for 24 h. (c) ROS levels in the treated HL-60 cells detected by flow cytometry. (d) CD11b expression. HL-60 cells were treated with the indicated compounds in the absence or presence of PKC inhibitor Gö 6983 (1 μM) for 72 h.
